# Dietary *Ficus carica* Inhibits Cognitive Impairment in Hypoxia-induced Non-alcoholic Fatty Liver Disease

**DOI:** 10.24546/0100495569

**Published:** 2025-04-14

**Authors:** DWI WIDYAWATI, ANDREANYTA MELIALA, SISWANTO, PARAMITA NARWIDINA, AYU TIARA FITRI

**Affiliations:** 1 Master Program of Department of Physiology, Faculty of Medicine, Public Health and Nursing, Universitas Gadjah Mada, Jalan Farmako, Sekip Utara, Yogyakarta, Indonesia; 2 Departement of Physiology, Faculty of Medicine, Public Health and Nursing, Universitas Gadjah Mada, Jalan Farmako, Sekip Utara, Yogyakarta, Indonesia; 3 Clinical Nutrition Research Group, Yogyakarta, Indonesia

**Keywords:** *Ficus carica*, Hypoxia, Non-alcoholic fatty liver disease, Cognitive impairment, Antioxidant

## Abstract

Intermittent hypoxia (IH) can aggravate non-alcoholic fatty liver disease (NAFLD) by activating hypoxia-inducible Factor-α and increasing oxidative stress. Lipid peroxidation, which occurs as a consequence of reactive oxygen species (ROS) generation, is characterized by malondialdehyde (MDA) formation and decreased catalase (CAT) and superoxide dismutase (SOD) levels. Hence, inadequate management of NAFLD might induce cognitive impairment. However, although studies have shown that *Ficus carica* could prevent cognitive impairment due to NAFLD complications, the mechanism by which this is achieved remains unclear. The current study therefore aimed to clarify the effects of *Ficus carica* in suppressing cognitive impairments caused by hypoxia-induced NAFLD. *Sprague-Dawley* male rats were divided into five groups: negative control positive control (PC), and IH with *Ficus carica* treatment (6.25, 12.5, and 25 mL/kg/day) for 4 weeks before and 1 week during IH. Rats were exposed to IH exposure by placing them in a hypoxic chamber (90% N_2_ and 10% O_2_) for 7 days. Regardless of dosage, *Ficus carica* treatment reduced MDA levels when compared to PC, and low-dose increased liver SOD levels more than the other groups. In contrast, the medium *Ficus carica* dose was associated with increased CAT activity and decreased inflammatory marker levels compared to the other treatments. Meanwhile, the neutrophil-to-lymphocyte ratio, platelet-to-lymphocyte ratio, and systemic immune-inflammation index were all greatest in the medium-dose group. The PC group showed a significant decrease in the percentage of alteration. Our data implies that medium doses of *Ficus carica* can reduce cognitive impairment caused by hypoxia-induced NAFLD.

## INTRODUCTION

Clinical and experimental evidence indicates that hypoxia significantly contributes to the pathophysiology of non-alcoholic fatty liver disease (NAFLD), with 25–35% of obese individuals suffering from recurrent apnea episodes during sleep, resulting in intermittent nocturnal hypoxia linked to all components of the metabolic syndrome, including NAFLD (1). The prevalence of obstructive sleep apnea (OSA) is estimated to be approximately 4–14% in Asian populations, significantly higher among obese individuals (2). This condition is linked to advanced liver fibrosis in patients with NAFLD, with fibrosis rates in Asian countries around 3–5% and in Western countries approximately 10% (3).

Hypoxia is a condition characterized by diminished oxygen availability and heightened oxygen utilization. Hypoxia can be categorized as acute or chronic based on the duration of the hypoxic episode. Both acute and chronic hypoxia may induce significant brain impairments. Despite substantial progress in the understanding of the pathophysiological mechanism of hypoxia, limited research has concentrated on cognitive dysfunction. Hypoxia can adversely affect various cognitive domains, including attention, learning and memory, processing speed, and executive function, with similarities having been observed in both acute and chronic hypoxia (4). The intensity of cognitive impairment has been associated with the length and extent of hypoxia. Although recovery is possible following acute hypoxia, persistent hypoxia may result in sequelae or even dementia, potentially due to distinct molecular pathways (5). A recent study discovered that cognitive dysfunction is a complication of NAFLD, a liver condition associated with metabolic syndrome, considering that 70% of NAFLD patients present with memory, attention, concentration, forgetfulness, and confusion, all of which negatively impact everyday living and quality of life (6).

IH may be a primary cause of NAFLD in animal models given that it directly promotes hepatosteatosis through repeated short episodes of hypoxia and reoxygenation (7). Low oxygen saturation is a risk factor that exacerbates the severity of NAFLD, and there is a possibility. IH exposure in the liver can alter the structure and function of mitochondria. Additionally, IH that causes oxidative stress can trigger pathological conditions in the liver that can disrupt its primary function as a metabolic center, subsequently affecting other organs within the body (8). Under normal conditions, reactive oxygen species (ROS) formation and antioxidant activity maintain a balance within the cells. However, a disruption in this equilibrium can trigger oxidative stress which promotes damage to cellular components, as evidenced by increased malondialdehyde (MDA). The human body possesses multiple antioxidant defense mechanisms, particularly through enzymatic systemic hypoxia induces oxidative damage to liver cells, as evidenced by elevated MDA levels and decreased activity of the antioxidant enzymes MnSOD and CAT. In addition to the involvement of inflammation in the development of hypoxia, there has been growing interest in using hematological indicators as systemic inflammation. Systemic immune-inflammatory biomarkers, such as the systemic immune-inflammatory index (SII), neutrophil to lymphocyte ratio (NLR), and platelet to lymphocyte ratio (PLR), were associated with risk and severity of various liver diseases; however, research on their role and clinical significance in metabolic disorders, particularly NAFLD, remains limited and findings are inconsistent (9).

Several studies have effectively demonstrated that systemic inflammation is a fundamental component of the gut-liver-brain axis and is a key contributor to the development of hepatic encephalopathy, a neuropsychiatric disease linked to increased liver injury (10). The enterohepatic axes link the liver to the gut, and under specific clinical situations, intestinal bacteria may migrate to the liver via the portal vein, resulting in aberrant immune system activation that triggers inflammatory reactions and damage. The consumption of dietary fiber, as a metabolite of bacterial fermentation, may inhibit the progression of NAFLD via the portal vein segment, which serves as a signaling molecule linking intestinal circumstances with physiological metabolism. Short-chain fatty acids (SCFA) can uphold intestinal barrier homeostasis and mucosal integrity, preventing certain toxic substances and inflammatory mediators from infiltrating the liver, thereby alleviating NAFLD (11). Subsequently, SCFA traverses the blood-brain barrier, mitigating neuroinflammation by enhancing neurogenesis, and serotonin biosynthesis, and improving homeostasis and neuronal function (12).

Present research has emphasized the consumption of natural foods rich in bioactive compounds, including polyphenols, flavonoids, and dietary fiber. *Ficus carica*, a herbal plant with antioxidant, contains total polyphenolics (37.0–463.0 mg of GAE/100g), total flavonoids (1.6–45.6 mg of catechin/100g), total anthocyanins (0.1–27.3 mg of cyn-3-Glu/1000g), and has a Trolox Equivalent Antioxidant Capacity (TEAC) value of 20.8–1987.0 μmol of TE/100g (13) and dietary fiber (9.32 g/100g) (14) content. This research attempts to explain the effects of IH exposure in triggering risk factors for OSA which in turn can cause NAFLD, which ultimately causes cognitive dysfunction by determining the role of functional foods as a source of antioxidants and dietary fiber. Indeed, evidence has shown that functional foods have the potential to counteract the negative impacts of oxidative stress caused by IH exposure, thereby preventing the emergence of more severe pathogenesis.

Oxidative stress and mitochondrial damage have been identified as the primary causes and contributors to NAFLD, which, if inadequately managed, may lead to cognitive impairment, including diminished concentration, ability to process information, and memory. Accordingly, the present study aimed to determine the effects of *Ficus carica*, in protecting the liver from inflammation and suppressing cognitive dysfunction. We hypothesized that ingesting *Ficus carica* may enhance the availability of antioxidants diminished by ROS formation resulting from hypoxia. Its dietary fiber content could ameliorate cognitive impairment via the gut-liver-brain axis repair mechanism.

## MATERIALS AND METHODS

### Preparation of whole *Ficus carica*

This study utilized *Ficus carica* of the Jordan variety cultivated in Bogor, West Java, Indonesia. Fresh *Ficus carica* was weighed, sliced, crushed, blended, and homogenized using an Armfield L4R (8033 rpm, 20 min) to produce a puree with a soluble solid content of 10.75 ± 0.070 Brix. It was subsequently refrigerated at −4°C until required.

### Animals and treatments

The Medical and Health Research Ethics Committee of the Faculty of Medicine, Public Health and Nursing, Universitas Gadjah Mada (approval number: KE/FK/0532/EC/2022; date: April 28, 2022) approved the study protocol, and the institution rules were followed for the care and use of laboratory animals. Twenty-four 15-week-old male *Sprague-Dawley* rats, weighing 250–300 g, were obtained from Central Food Nutrition Study, Universitas Gadjah Mada. The animals were housed under a controlled temperature of 25°C and a 12-h/12-h light/dark cycle. The rats were fed standard laboratory chow and water *ad libitum*.

The sample size in this study was calculated using the Federer formula as follows: (n − 1) (k − 1) ≥ 15;

where:

n: number of samples per group
k: number of groups
(n − 1) (5 − 1) ≥ 15
4n − 4 ≥ 15
n ≥ 4.75

**Figure 1:**
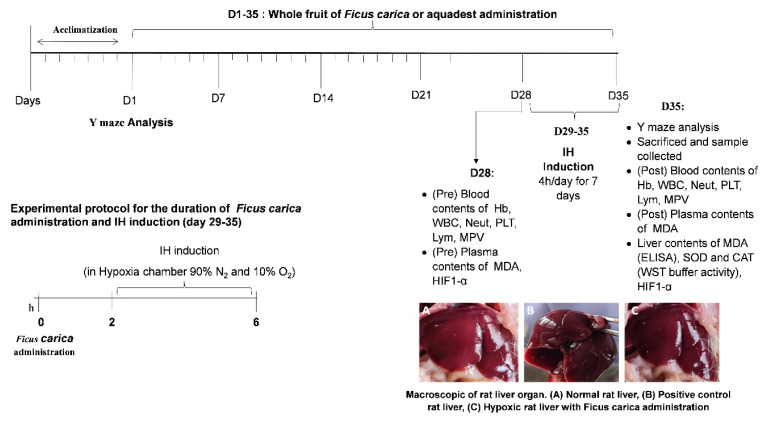
Experimental protocol

The minimum sample for this study, per group, is 4.75 rounded up to 5. There was an anticipation of dropping out, the number of rats in each group was 6, so the total number of rats was 24. The sampling method was simple random sampling consisting of five groups, twenty-four male rats were divided into five groups: the negative control group (NC, fed standard food without IH exposure, N = 6), positive control group (PC, administered distilled water and exposed to IH, N = 5), and three groups of rats exposed to IH and administered whole *Ficus carica* (WF) at a low (6.25 ml/kg/day, IH+Low WF group, N = 6), medium (12.5 mg/kg/day, IH+Med WF group, N = 6), or high dose (25 ml/kg/day, IH+High WF group, N = 6). The groups originally had six rats; however, following the adaptation phase, the rats were unwell and were consequently omitted from the study. In the WF groups, *Ficus carica* was administered for 4 weeks before the induction of IH and during the 1 week of IH exposure. The experimental design of this research is presented in Figure 1. The hypoxia induction equipment and procedures used in this study were based on previous research (14). The gas mixing set initially contained 5 L of water. It was determined that the oxygen level in the gas mixing set was 10%, and the nitrogen level was 90% applied in the morning (8 a.m.–1 p.m.). This determination was made when the water in the gas mixing set was completely depleted. This is based on the principle that when air enters a space filled with water, it displaces it. The hypoxic protocol consisted of 7 consecutive days of exposure to IH. The rats were subjected to hypoxia by being placed in a transparent chamber connected to a gas tank containing a mixture of 10% oxygen and 90% nitrogen. Subsequently, the rats were transferred to the counting chamber. If the rat appears too weak, it will be promptly evacuated from the hypoxic chamber to access fresh air. Should the condition improve, he will proceed with the research. The anticipated outcome of this study is that the group of rats receiving *Ficus carica* will successfully endure 7 days of intermittent hypoxia without complications, maintaining their health, whereas the group not consuming *Ficus carica* will survive, allowing for subsequent cognitive behavior assessment via the Y maze and facilitating blood sampling.

Initially, the oxygen level inside and outside the chamber was precisely measured using an oximeter. Once the accuracy was confirmed, a rat was placed inside the chamber. Subsequently, a gas mixture was introduced into the chamber using a gas mixing set until the oxygen level reached 10–13%, as indicated by the oximeter. Oxygen levels in the chamber were measured at 30-minute intervals for 4 hours on a single day. The design of the hypoxic chamber and gas mixing set employed in this study was based on prior research (14).

### Y-maze test

The Y-Maze is a behavioral technique employed to assess cognitive function in rats, specifically utilized to evaluate their short-term spatial memory as a type of recognition. Spatial memory scores were evaluated 1 week before treatment and on the 35th day of the experiment using the Y-maze test, as previously documented (15), in the Behavioural Testing Room of the Department of Physiology, Faculty of Medicine, Public Health and Nursing, Universitas Gadah Mada. Rats were positioned at the convergence of the three arms of the Y labyrinth and let to make their arm selections for a total length of 5 minutes, each arm was 50 cm long, 20 cm high, and 10 cm wide (15). The frequency of accurate decisions/alterations (ABC, ACB, BCA, BAC, CBA, or CAB) among the arms (ABC) was documented to ascertain the memory index (% of correct alteration). The maze was sanitized with 70% ethanol between trials to remove smell clues. The % alternation was computed as follows:


$$\text{Alternation }(\%)=\frac{\text{Number of alternation}}{\left(\text{Total arms entry}-2\right)}\times 100\%$$

### Biochemical analysis of oxidative stress and inflammation markers

Approximately 2 ml of blood was obtained from the retroorbital veins and collected in EDTA-coated vacutainer tubes (BD Bioscience, Franklin Lakes, NJ, USA). An EDTA test was performed for routine hematological profiling using a fully automated hematology analyzer (Sysmex® XP-100). These indices are essential instruments for evaluating systemic inflammation and have been utilized in prior NAFLD investigations as follows (9):

NLR (neutrophil-to-lymphocyte ratio): N/L.
PLR (platelet-to-lymphocyte ratio): p/L.
Systemic immune-inflammation index (SII): P × N/L.

Furthermore, vacutainer tubes were centrifuged at 1200 g for 25 minutes at 4°C for plasma separation, utilizing a Sigma 3-30KS superspeed chilled centrifuge. Fresh livers were weighed, rinsed with 0.9% NaCl, frozen in liquid nitrogen, and stored at −80°C for subsequent analysis. Plasma was utilized to assess corticosterone, serotonin, and HIF-1α levels. Liver tissue was used for the assessment of SOD, CAT, and MDA concentrations.

ELISA Kits were used to determine plasma and brain HIF-1α and HIF-2α levels in the control and treatment groups. Rat HIF-1α (Hypoxia-inducible factor 1-alpha) ELISA Kit (FINETEST, Catalog No. ER0191-96 well) and Rat HIF-2α (FINETEST, Catalog No. ER2001) are available. Each kit’s protocol was followed for determination. Serotonin and cortisol levels were assessed with ELISA kits with the subsequent specifications. 5-Hydroxytryptamine ELISA Kit (5-HT), 96 wells, ABCLONAL, Catalog No. RK00607, and Corticosterone ELISA Kit, 96 wells, ABCLONAL, Catalog No. RK09054. The protocol of each kit conducted the measurements.

The CheKine™ Micro Lipid Peroxidation (MDA) Assay Kit (KTB1050) quantified MDA in plasma samples. The quantification of lipid peroxide involved a colorimetric reaction using thiobarbituric acid, as described by Janero. Lipid peroxide levels were determined by calculating the MDA level (16).

SOD activity was measured as described previously (17, 18). The procedure and data analysis were performed according to the instructions provided by the manufacturer (SOD Assay Kit-WST, Dojindo Laboratories ASIA). The optical density at 450 nm was determined by using a microplate reader (epoch2, BioTek Instruments). Blank 1 represented the coloring without inhibitor, blank 2 represented the sample blank, and blank 3 represented the reagent blank. SOD activity was determined using the following formula:


SOD activity (inhibition rate %)=[(Ablank 1-Ablank 3)-(Asample-Ablank 2)]/(Ablank 1-Ablank 3)×100.

According to the instructions, a single unit of SOD was determined as the quantity of the enzyme present in a 20-μl sample solution that reduced the reaction of WST-1 with superoxide anion by 50%.

CAT activity was measured using a previously outlined procedure (19) with minor adjustments. CAT activity was measured according to the reduction of hydrogen peroxide. Liver supernatant (0.5 ml) was combined with 2.0 ml of 50 mM potassium phosphate buffer (pH 7.0) in a quartz cuvette containing 10 mM hydrogen peroxide. Absorbance changes were quantified using a UV-vis spectrophotometer at a specific wavelength of 240 nm, with measurements taken every 15 s over 1 min. CAT activity was calculated by determining the slope of the absorbance curve for both the sample solution (SL) and the blank solution (SLb) using the following equation: CAT activity (U/mg) = (SL − SLb)/0.436 × 2.5/0.5.

### Statistical Analysis

Statistical analysis was conducted to determine the difference in statistical significance among the groups. Graph Pad Prism version 10.0 was utilized for analysis, with p-values below 0.05 considered statistically significant. Analysis of variance (ANOVA) was employed to compare the groups. Data were expressed as Mean ± Standard Deviation (SD).

## RESULTS

### Effect of whole *Ficus carica* on body and liver weight

All of the rats subjected to hypoxia exhibited weight loss, with the PC group demonstrating the most significant percentage of decrease in weight, significantly differing from the NC and IH+Med WF groups. The liver weight in the IH-induced group was generally lower than that of the NC group; however, significant differences were observed only in the IH+Low WF group, whereas the IH+High WF group exhibited average liver weight and relative liver weight that were not significantly different from the NC group. Regarding the comparative liver weights, no significant difference was seen among the PC, IH+Low WF, and IH+High WF groups (Table I).

### Effect of whole *Ficus carica* on HIF1-α in plasma and brain

**Figure 2:**
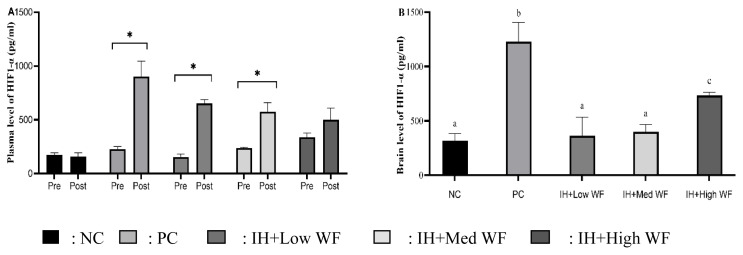
Effect of whole *Ficus carica* on HIF1-α in plasma and brain *p < 0.05 compared to pre and post (A). Different letters (a,b,c) in the same column indicate a significant difference between groups according to the one-way ANOVA (p < 0.05) and Duncan post-hoc test.

The present study examined the concentrations of HIF1-α in rats’ plasma and brain tissue. As illustrated in Figure 2A, the PC, IH+Low WF, and IH+High WF groups exhibited a significant elevation in HIF1-α levels before and after WF administration and IH exposure. Figure 2B indicates that HIF-1α levels in the PC group’s brain were significantly higher than in the other groups. The NC, IH+Low WF, and IH+Med WF groups exhibited no significant differences. In contrast, the high WF administration group demonstrated increased levels relative to the NC, IH+Low WF, and IH+Med WF groups, yet significantly lower than the PC group.

### Effect of whole *Ficus carica* on anti-inflammatory as NAFLD parameters

**Figure 3:**
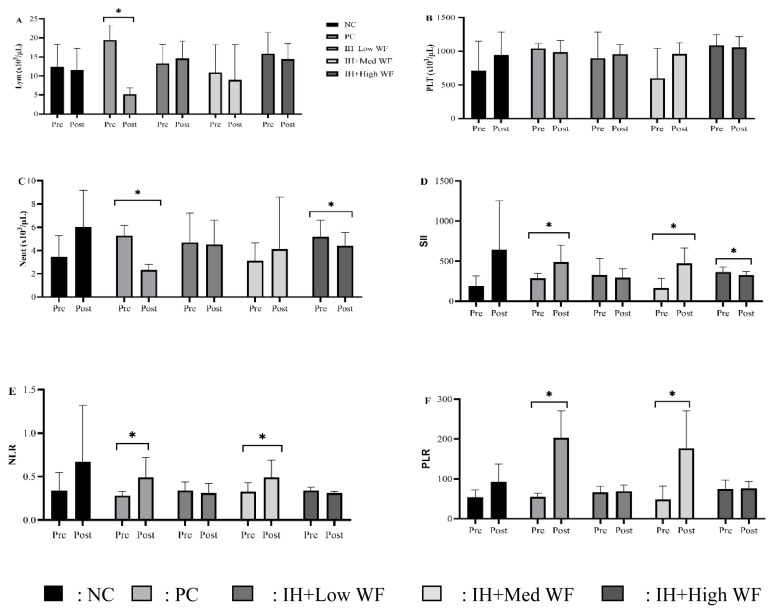
Effect of whole *Ficus carica* on anti-inflammatory as NAFLD parameters Lym, Lymphocyte; NEUT, Neutrophil; SII, systemic immune-inflammation index; NLR, neutrophil-to-lymphocyte ratio; PLR, platelet-to-lymphocyte ratio. *p < 0.05 compared to pre and post.

In the inflammatory markers in blood among experimental groups in Figure 3, neutrophil counts significantly differed between the PC and IH+High WF groups, with exposure to IH resulting in considerably decreased neutrophil counts compared with the pre-IH level. Platelet counts did not differ between before and after IH in any group. Previously, multiple observations indicated that hypoxic circumstances have a detrimental effect on the immune system. We employed inflammation markers such as PLR, NLR, and SII to comprehend this phenomenon comprehensively. After the induction of IH, significant elevation of NLR, PLR, and SII was observed in both the PC and IH+Med WF groups compared with their respective values before IH induction. In the IH+High WF group, only SII significantly differed between before and after IH exposure.

### Effect of whole *Ficus carica* on antioxidant enzyme activity and lipid peroxidation in liver

**Figure 4:**
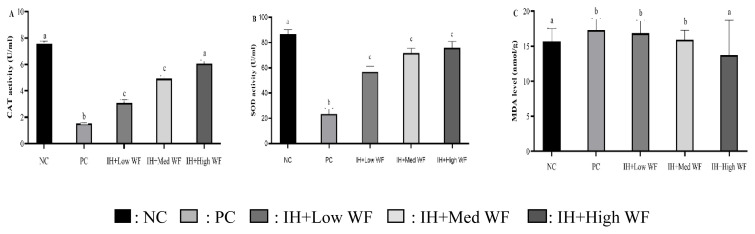
Effect of whole *Ficus carica* on antioxidant enzyme activity and lipid peroxidation in the liver Different letters indicate a significant difference between groups according to the one-way ANOVA (p < 0.05) and Duncan post-hoc test. MDA, Malondialdehyde; CAT, Catalase; SOD, Superoxide Dismutase; WF, whole of Ficus carica.

IH exposure induced a significant reduction in the antioxidant enzymatic activity like SOD and CAT, and *Ficus carica* administration in all doses showed a significant increase in SOD activity in comparison with PC group (Figure 4). The activity of lipid peroxidation was found to be significantly higher in the liver of rats exposed to IH in comparison to the NC group, except in IH+High WF not significantly significant in comparison to the NC group.

### Effect of the whole *Ficus carica* on memory performance assessment with Y-maze test

**Figure 5:**
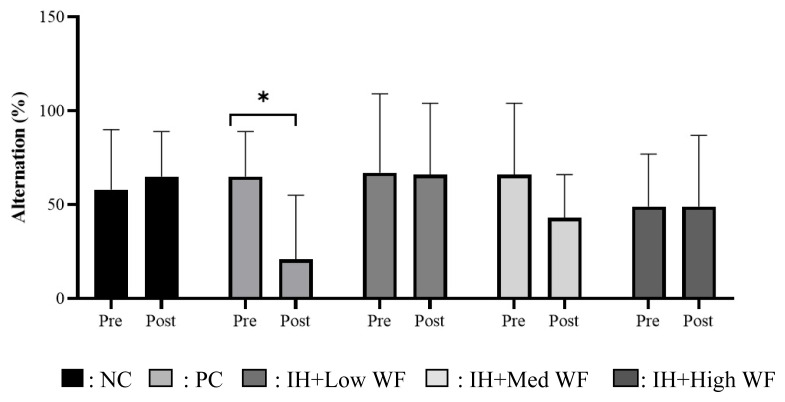
Effect of the whole *Ficus carica* on memory performance assessment with Y-maze test *p < 0.05 compared to pre and post.

The Y-maze memory performance assessment result showed a significant decrease only in the PC group, and there was no significant difference observed in the percentage of alternation assessed from right/wrong decisions scored pre- and post-test in the IH group across all doses and the NC group (Figure 5).

### Correlations between behavioral parameters, antioxidant, and inflammation parameters in the liver as NAFLD marker

The data of the correlations between spatial memory performance with antioxidant enzyme activity in the liver and inflammation parameters in the blood are shown in Table II. PLT had a significant positive correlation with liver MDA levels, while the SII parameter index demonstrated a significant negative correlation with Lym. This study found a negative correlation between spatial memory performance and SOD enzyme activity in the liver and Lym levels in the blood (significant; p < 0.05).

## DISCUSSIONS

**Figure 6:**
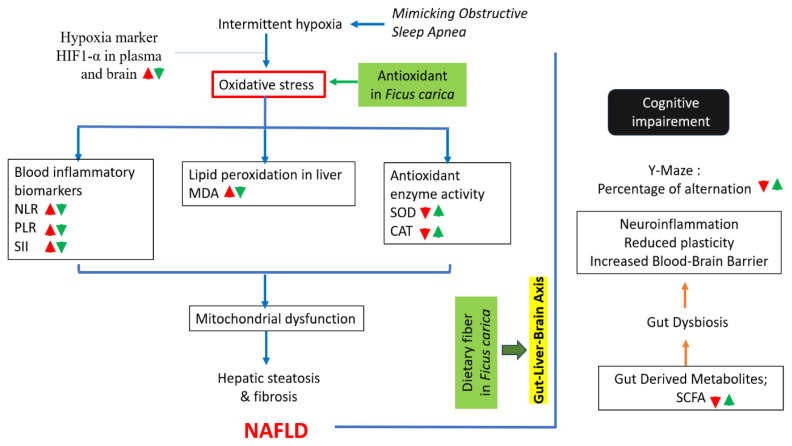
Graphical summary of the discussion illustrating the advantageous administration of *Ficus carica* Increase 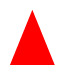 or decrease 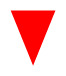 impact of oxidative stress condition Increase 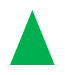 or decrease 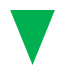 action of *Ficus carica* administration

Current research indicates that OSA is linked to the onset and progression of NAFLD, irrespective of obesity and other risk factors for illness. The correlation between NAFLD and OSA is linked to the extent of nocturnal hypoxemia in OSA; hence, the majority of animal models have concentrated on inducing intermittent hypoxia, the primary characteristic of OSA, to clarify the pathways via which OSA contributes to the intricate metabolic disorders observed in NAFLD (7). The correlation between NAFLD and metabolic syndrome emphasizes cognitive dysfunction, including issues with memory, attention, and concentration, leading to the notion of metabolic cognitive syndrome related to NAFLD; nevertheless, the underlying mechanism remains ambiguous. It was shown in Figure 6.

Hypoxia and inflammation are closely related, as inflammation can be induced by hypoxia or it can manifest during hypoxic conditions. Following induced hypoxia, molecular alterations indicate systemic inflammation, as demonstrated by an elevation in inflammatory protein levels in plasma within 1 h of hypoxia (20). The relative liver weight is a prognostic indicator for alterations in biochemical activity that contributes to maintaining optimal function, including food intake. Exposure to hypoxia influences lipid and glucose metabolism, resulting in reduced appetite and weight reduction. The mechanisms of hypoxia might induce weight loss through: 1) decreased appetite, as hypoxia inhibits appetite-regulating hormones like ghrelin and elevates anorexigenic substances such as leptin, glucagon-like peptide-1 (GLP-1), peptide YY (PYY), and cholecystokinin (CCK) (21), 2) increased energy expenditure and hypoxia elevate the basal metabolic rate and enhance fat oxidation (22), 3) enhanced fatty acid consumption; hypoxia can augment fatty acid utilization for energy production (23), 4) intermediary in protein catabolism; hypoxia can stimulate protein catabolism to fulfill energy requirements (24). The inclusion of *Ficus carica* in the diet before and during IH exposure led to an increase in liver weight. However, there were no significant differences in the absolute and relative liver weights compared with the findings in the absence of *Ficus carica* administration and IH exposure. This finding aligns with previous research reporting a correlation between IH and changes in body weight. These changes are believed to result from the presence of HIF-α, which is induced by the body’s adaptive response to low oxygen levels (25).

Inflammation can trigger the activation of genes in the HIF pathway, whereas hypoxia can regulate inflammatory signals. Although these molecular pathways can work together, the physiological effects of inflammation are caused by hypoxia (26). HIF1-α is a transcription factor that is a principal regulator of cellular responses, particularly in brain tissue during cerebral hypoxia and ischemia and is implicated in various activities, including metabolism, proliferation, and angiogenesis. HIF-1α may exert both advantageous and adverse effects on the brain, contingent upon the intensity and duration of hypoxia exposure. The presence of HIF1-α in the brain confers protection against hypoxic-ischemic injury by mitigating inflammation and decreasing the activation of astrocytes and microglia, while also serving as a crucial regulator of hippocampal neurogenesis, facilitating learning and memory (27). Prolonged hypoxic exposure can render HIF-1α detrimental to the brain by inducing neurovascular inflammation, oxidative stress, and cellular apoptosis. Furthermore, HIF-1α may exacerbate secondary brain injury by promoting neuroinflammation, specifically through inflammasome activation, mitochondrial dysfunction, and cell death (28).

The mechanisms by which hypoxia activates inflammatory signaling and immune function are not thoroughly known (29). Conversely, inflammation caused by hypoxia can have a beneficial effect by triggering immunological responses and facilitating the repair of tissues, particularly in cases of long-term oxygen deprivation. Neutrophils and lymphocytes have a crucial function in the inflammatory response by releasing different inflammatory mediators. NLR as an inflammatory marker offers the benefit of being a ratio that combines two distinct immune pathways, making it more effective than individual leukocyte indicators such as neutrophil, lymphocyte, and total leukocyte counts (30). NLR is frequently employed as a novel indicator of inflammation associated with chronic illnesses in stability findings. Exposure to hypoxia along with carbon monoxide exposure has positive correlations with the levels of carboxyhemoglobin, NLR, and PLR (26). As the primary effector cells of the innate immune response, neutrophils are essential for survival and proper functioning in challenging environmental conditions, such as hypoxia. The primary determinant of the functional lifespan of neutrophils is their capacity to undergo.

This study employed a 4-hour IH exposure method over 7 days, which has been demonstrated to induce liver tissue hypoxia, as indicated by elevated levels of HIF-1α in response to cellular hypoxia. This activation subsequently triggers genes that enhance oxygen supply and diminish expenditure, serving as a marker of NAFLD in this investigation through the analysis of inflammatory parameters in the blood, antioxidant enzyme activity, and MDA levels in the liver. SOD and CAT are the primary antioxidant enzymes that are crucial in neutralizing reactive oxygen species and mitigating oxidative stress. *Ficus carica* enhances SOD and CAT activities in the liver of IH-induced mice across all dosage levels. The CAT activity in mice treated with *Ficus carica* at a dosage of 25 ml/kgbw-1 exhibited no significant difference compared to the control group. The liver’s abundance of mitochondria renders it a primary target for damage inflicted by reactive oxygen species, the synthesis of which remains excessive due to ischemia-hypoxia induction (31), Hypoxia exposure may serve as a precipitating factor for NAFLD. MDA is a frequently utilized indication of elevated fatty liver index, serving as a marker for NAFLD (32), where modifications to eating habits aid in diminishing levels of inflammatory markers, particularly in NAFLD circumstances.

The elevation of MDA levels in the IH-exposed group without *Ficus carica* treatment is probably due to the release of substantial quantities of superoxide radicals by liver granuloma macrophages. Apoptosis is inhibited by hypoxia in both humans and animals (33). Neutrophil apoptosis is a regulated cellular demise mechanism that inhibits the harmful release of neutrophils.

Flavonoids in *Ficus carica* have been found to enhance the immune system, as demonstrated by an *in vitro* study of macrophage phagocytic activity and lymphocyte proliferation. The research concluded that *Ficus carica* contains a total flavonoid content of 0.74 ± 0.01 mgQE/g, indicating its potential as an immunostimulant against macrophage phagocytic activity (34). Nevertheless, it does not influence lymphocyte proliferation. Our research findings indicate that exposure to 4 hours of intermittent hypoxia for 7 consecutive days can decrease superoxide dismutase and catalase activity compared to observations under normoxic conditions. Nonetheless, WF can mitigate the adverse impacts of these declines. This is likely attributable to the antioxidant properties of WF, which can effectively neutralize the excessive production of free radicals during IH exposure. This is supported by the MDA level change, which reflects lipid peroxidation. Rats treated with varied doses of WF exhibited similar MDA levels as NC rats. In response to oxidative stress induced by IH exposure, the body relies on a combination of internal and external sources of antioxidants to eliminate ROS and decrease MDA levels (35, 36). The findings of this study suggest that *Ficus carica* possesses antioxidant properties and the ability to serve as a source of external antioxidants. These antioxidants can assist the body in protecting against free radicals generated by exposure to IH. Moreover, the levels of MDA are inversely associated with those of antioxidants. It has been verified that *Ficus carica* contains polyphenols and flavonoids (37), as well as anthocyanins, which exhibit significant antioxidant activity (13), Cyanidin-3-O-rutinoside is the primary anthocyanin found in *Ficus carica*, comprising 92% of the total antioxidant capacity of the anthocyanin fraction.

Increased oxidative stress, indicated by high MDA levels in the group subjected to IH induction without *Ficus carica* administration, substantiates that oxidative stress significantly contributes to the pathophysiology of chronic inflammatory liver conditions, including NAFLD. This chronic disorder of lipid metabolism is intricately linked to alterations in the oxidant/antioxidant equilibrium, which impacts metabolism-related organelles, resulting in cellular lipotoxicity, lipid peroxidation, persistent endoplasmic reticulum stress, and the onset of mitochondrial dysfunction (38). Controlling the endogenous antioxidant response by consuming exogenous antioxidants from dietary sources, such as *Ficus carica*, is a compelling strategy for preventing the onset of NAFLD. Rats subjected to intermittent hypoxia without *Ficus carica* treatment exhibited elevated MDA levels and diminished SOD and CAT activity in the liver, indicative of NAFLD. Additionally, these rats demonstrated a marked reduction in alternation rates, signifying impaired spatial working memory, as evidenced by a significant decrease in arm entries during the Y-maze test relative to other groups. *Ficus carica* possesses a substantial concentration of vitamins, amino acids, and antioxidant substances that have neuroprotective properties, while its quercetin content aids in alleviating cognitive impairments associated with Alzheimer’s disease (39).

This study presents evidence endorsing *Ficus carica* as a source of antioxidants in preventive strategies against the effects of IH, which may promote NAFLD and, if unregulated, result in cognitive impairment. This study demonstrates that *Ficus carica* may safeguard spatial working memory following exposure to intermittent hypoxia, suggesting potential advancements in the treatment of memory impairment associated with oxidative stress. This study was limited to behavior, hypoxia marker (HIF1-α in plasma and brain), oxidative stress markers in the liver (SOD, MDA, and CAT), and blood inflammatory markers (NLR, PLR, SII) when exploring the effects of *Ficus carica* on IH-exposed rats. Liver and brain histopathology and liver function parameters (AST, ALT, total bilirubin, and γGTP) are also affected by IH-induced NAFLD, which should be explored in future studies.

## Figures and Tables

**Table I:** Absolute liver weight and relative liver weight among experimental groups

Group	Body weight (g)			Absolute liver weight (g)	Relative liver weight (%)**

	Initial	Final	Percentage change		
NC	256.33 ± 20.00^a^	268.33 ± 12.22^a^	4.86 ± 3.52^a^	10.35 ± 1.04^ab^	3.99 ± 1.92^a^
PC	283.00 ± 35.16^a^	262.40 ± 30.37^a^	−7.19 ± 2.34^b^	8.88 ± 1.39^bc^	3.38 ± 0.17^bc^
IH+Low WF	271.00 ± 26.88^a^	255.00 ± 29.38^a^	−6.00 ± 2.75^b^	8.46 ± 1.08^c^	3.32 ± 0.18^c^
IH+Med WF	282.00 ± 33.11^a^	277.50 ± 30.57^a^	−1.50 ± 2.76^c^	10.19 ± 2.13^abc^	3.65 ± 0.37^b^
IH+High WF	284.17 ± 17.10^a^	265.50 ± 14.43^a^	−6.52 ± 1.92^b^	10.94 ± 0.92^a^	4.12 ± 0.20^a^

** Relative liver weight = Liver weight (g)/Body weight (g) × 100%.

Values are expressed as Mean ± SD. Values with different superscriptions within the same columns significantly differ at P < 0.05. IH, Intermittent Hypoxia; WF, whole *Ficus carica*; NC, Negative control; PC, Positive control; Low WF: 6.25 ml/kg/day, Med WF: 12.5 ml/kg/day; High WF: 25 ml/kg/day. Values are expressed as Mean ± SD. Different letters (a, b, c) in the same column indicate a significant difference between groups according to the one-way ANOVA (p < 0.05) and Duncan post-hoc test. WF, whole of *Ficus carica*.

**Table II:** Correlations between antioxidant, inflammation, and behavioral parameters in the liver as NAFLD marker

		SOD	MDA	CAT	Lym	PLT	NEUT	SII	NLR	PLR	Ymaze
Antioxidant parameters in the liver	SOD										
	MDA	−0.021									
	CAT	−8.66	−3.09								
										
Inflammation parameter	Lym	0.433	−0.296	−0.600							
	PLT	0.153	0.933*	−0.373	−0.387						
	NEUT	0.138	−0.307	0.308	−0.530	−0.212					
	SII	−0.093	0.291	0.345	−0.911*	0.398	0.753				
	NLR	−0.225	−0.126	0.598	−0.838	−0.035	−0.894*	0.900*			
	PLR	−0.253	0.652	0.279	−0.909*	0.711	0.360	0.883*	0.641		
										
Behavioral parameter	Ymaze	−0.191	0.174	0.439	−0.924*	0.393	0.517	0.856	0.777	0.812	

Values represent the Pearson correlation coefficient and r. values in bold letters show significant correlations (p < 0.05). SOD, Superoxide Dismutase; MDA, Malondialdehyde; CAT, Catalase; Lym, Lymphocyte; NEUT, Neutrophil; SII, systemic immune-inflammation index; NLR, neutrophil-to-lymphocyte ratio; PLR, platelet-to-lymphocyte ratio.
